# Translation, Cultural Adaptation, and Reproducibility of the Physical Activity Readiness Questionnaire for Everyone (PAR-Q+): The Brazilian Portuguese Version

**DOI:** 10.3389/fcvm.2021.712696

**Published:** 2021-07-26

**Authors:** Juliano Schwartz, Paul Oh, Monica Y. Takito, Bryan Saunders, Eimear Dolan, Emerson Franchini, Ryan E. Rhodes, Shannon S. D. Bredin, Josye P. Coelho, Pedro dos Santos, Melina Mazzuco, Darren E. R. Warburton

**Affiliations:** ^1^Physical Activity Promotion and Chronic Disease Prevention Unit, University of British Columbia, Vancouver, BC, Canada; ^2^Cardiovascular Prevention and Rehabilitation Program, Toronto Rehabilitation Institute, University Health Network, Toronto, ON, Canada; ^3^Department of Pedagogy of the Human Body Movement, School of Physical Education and Sport, University of São Paulo, São Paulo, Brazil; ^4^Applied Physiology and Nutrition Research Group, School of Physical Education and Sport, Rheumatology Division, Faculdade de Medicina FMUSP, University of São Paulo, São Paulo, Brazil; ^5^Institute of Orthopaedics and Traumatology, Faculty of Medicine FMUSP, University of São Paulo, São Paulo, Brazil; ^6^Sport Department, School of Physical Education and Sport, University of São Paulo, São Paulo, Brazil; ^7^School of Exercise Science, Physical and Health Education, University of Victoria, Victoria, BC, Canada; ^8^Association for Assistance of Disabled Children, São Paulo, Brazil; ^9^Department of French, Hispanic and Italian Studies, University of British Columbia, Vancouver, BC, Canada; ^10^Aurora Physio & Care, Physiotherapy Center, Campinas, Brazil

**Keywords:** health, exercise, cardiovascular disease, physical activity, risk stratification, translation

## Abstract

**Background:** The Physical Activity Readiness Questionnaire for Everyone (PAR-Q+) is the international standard for pre-participation risk stratification and screening. In order to provide a practical and valid screening tool to facilitate safe engagement in physical activity and fitness assessments for the Brazilian population, this study aimed to translate, culturally adapt, and verify the reproducibility of the evidence-based PAR-Q+ to the Brazilian Portuguese language.

**Method:** Initially, the document was translated by two independent translators, before Brazilian experts in health and physical activity evaluated the translations and produced a common initial version. Next, two English native speakers, fluent in Brazilian Portuguese and accustomed to the local culture, back-translated the questionnaire. These back translations were assessed by the organization in charge of the PAR-Q+, then a final Brazilian version was approved. A total of 493 Brazilians between 5 and 93 yr (39.9 ± 25.4 yr), 59% female, with varying levels of health and physical activity, completed the questionnaire twice, in person or online, 1–2 weeks apart. Cronbach's alpha was used to calculate the internal consistency of all items of the questionnaire, and the Kappa statistic was used to assess the individual reproducibility of each item of the document. Additionally, the intraclass correlation coefficient and its 95% confidence interval (CI) were used to verify the general reproducibility (reliability) of the translated version.

**Results:** The Brazilian version had an excellent internal consistency (0.993), with an almost perfect agreement in 93.8% of the questions, and a substantial agreement in the other 6.2%. The translated version also had a good to excellent total reproducibility (0.901, 95% CI: 0.887–0.914).

**Conclusion:** The results show this translation is a valid and reliable screening tool, which may facilitate a larger number of Brazilians to start or increase physical activity participation in a safe manner.

## Introduction

Physical inactivity is related to several health problems and is estimated to lead to the premature death of ~9% of the global population (1). Routine physical activity participation protects against more than 25 chronic medical conditions, such as cardiovascular disease, diabetes, some types of cancer, depression, and osteoporosis, and significantly reduces the risk of early mortality (2, 3). Owing to these benefits, governments and health organizations around the world are investing in initiatives for the promotion of regular physical activity, including changes in the physical environment and public policies (4). However, there are other factors associated with physical inactivity, such as biological and psychological aspects (3, 5). In this respect, the fear of injury, getting sick, and even dying are reported, among others, as some of the most common barriers to physical activity (6–8). These concerns are shared by health professionals who prescribe supervised as well as unsupervised physical activity and exercise to prevent and manage chronic diseases (8–10).

To address this issue the Physical Activity Readiness Questionnaire (PAR-Q) was created in Canada in the 1970s, as a pre-participation screening tool, based on experts' opinion (11, 12). With seven health-related questions to be answered as Yes or No, the document was used extensively globally (13). However, various limitations to the survey were acknowledged (14–16). For instance, a major limitation of the PAR-Q was that its use was restricted to people between 15 and 69 years of age (17). Age restrictions on a front-line pre-participation screening tool is a contemporary issue for physical activity participation given population aging worldwide (18). Another significant limitation was the conservative nature of the PAR-Q, which led to many false positives (19). When an individual answered Yes to one or more questions, they were advised to consult a physician for clearance to participate in physical activity (20, 21). However, obtaining physical activity clearance from a physician may not be feasible in several jurisdictions (22). On a global scale, access to medical professionals can involve very long waiting lists for public services, and access to private options is limited and unaffordable for many (23, 24).

Given such limitations, a series of systematic reviews together with an evidence-based consensus process were performed to establish best practices in risk stratification for physical activity participation (25–34). From this process, a new, evidence-based pre-participation screening tool was created: The Physical Activity Readiness Questionnaire for Everyone (PAR-Q+) (20). In the current PAR-Q+, when the respondent answers No to all seven evidence-informed initial questions, they are self-cleared for unrestricted physical activity participation (35). If the individual answers Yes to one or more of these general health questions, they are required to complete follow-up questions on specific chronic medical conditions. If the individual responds No to all follow-up items, they are cleared to become more physically active. If a respondent answers Yes to one or more of these supplementary questions, they are referred to the electronic Physical Activity Readiness Medical Examination (ePARmed-X+; www.eparmedx.com), or to consult with a health professional qualified to prescribe exercise (19). Through this screening process, the vast majority of participants are able to self-clear for physical activity or exercise (36). Also, while the document has a total of 48 items, completing the tool is straightforward and takes ~5 min (19). Additionally, this new questionnaire was recently published in a digital format, thus providing the advantage of online completion (37).

Although chronic medical conditions are the primary cause of mortality in high-income countries, such as in Canada, these diseases affect low- and middle-income countries in a much higher proportion, with more than 75% of worldwide deaths from such diseases occurring in these nations (38, 39). This is the case of Brazil, a middle-income country, where chronic diseases are also a leading health problem (40). Before the COVID-19 pandemic, the main causes of deaths in the country were cancer and cardiovascular disease, which are directly linked to obesity (41, 42). The prevalence of obesity has been dramatically increasing in the country, with 12% of the youth population, as well as 20% of adults and 21% of older adults being considered obese in Brazil (43, 44). In large part, this scenario is due to sedentary lifestyles (45). Physical inactivity, a preeminent behavioral risk factor for chronic diseases and early mortality, is one of the most prevalent unhealthy behaviors in Brazilians (46, 47). According to studies about perceived barriers to physical activity in Brazil, having a disease and being afraid of getting injured are also among the main reasons preventing minors, adults, and the elderly from becoming more physically active (48–50). The country has one of the world's fastest aging populations, and over 70% of Brazilian seniors are considered insufficiently active (51, 52). This prevalence is 61% for adults (44) and over 80% for children and adolescents in the country (53).

Since the fear of worsening their health condition is among the main barriers to physical activity in Brazilians, an instrument like the PAR-Q+, validated to Brazilian Portuguese, could be crucial to allow numerous individuals to safely start or increase physical activity participation. Accordingly, the purpose of this study was to translate, culturally adapt, and verify the reproducibility of the questionnaire to the Brazilian context.

## Methods

This study was designed in two phases. Initially, the questionnaire was translated and culturally adapted to the targeted language. Subsequently, a test re-test procedure was adopted with different age groups to verify reproducibility.

## Translation and Adaptation

Permission to develop the Brazilian Portuguese version of the document was granted from the organization in charge of the questionnaire (i.e., the PAR-Q+ Collaboration). The screening tool was first translated into Brazilian Portuguese by two independent translators who speak Brazilian Portuguese as their native language. A group of Brazilian experts in health and physical activity then came together to produce a combined initial version. Overall, the experts involved in validating the PAR-Q+ in Brazilian Portuguese agreed with the translators' versions. Only a couple of minor phrasing adjustments were necessary to culturally adapt the PAR-Q+ to the Brazilian context. The next step was for two native English speakers, fluent in Brazilian Portuguese and accustomed to the Brazilian culture, with no previous exposure to the original PAR-Q+, to back-translate the questionnaire into English. When assessing these back-translations, the PAR-Q+ Collaboration noted a few terms slightly different from the original document. These were considered to have occurred due to the choice of terms in Brazilian Portuguese to allow a better understanding of the questionnaire by the Brazilian population, and these adaptations did not modify the original meaning. After having its accuracy ensured, a final version was approved (see Appendix 1).

## Field Testing

To assess the reproducibility of the translated version, Brazilians living in Brazil and abroad responded to the questionnaire on two separate occasions, 1–2 weeks apart. A total of 567 individuals attending health and fitness facilities as well as members from the general public, male and female from all age groups, were invited to take part in this project. There were no exclusion criteria. However, 74 individuals did not complete the questionnaire for the second time, mainly due to schedule incompatibility. Therefore, the sample was composed of 493 participants (59% female), between 5 and 93 years old (39.9 ± 25.4 yr). A total of 114 were children and adolescents, 252 were adults, and 127 were older adults. The questionnaire was administered in person to 84 individuals in a lifestyle management program focusing on chronic disease prevention, 11 clients at a physiotherapy clinic, 24 participants of a fitness project, 14 members of a CrossFit gym, and 43 patients from a rehabilitation center. The remaining 317 questionnaires were completed online. Respondents represented a variety of health status cohorts such as clinical populations, healthy individuals, athletes, and non-competitive exercisers. As per the guidelines of the PAR-Q+, those under the legal age had the questionnaire completed by their parents/guardians. In all settings and forms of application the participants were welcomed to provide comments, if any, about their understanding of the document. Participants also reported the time taken to answer the questionnaire for the first time.

### Statistical Analysis

Data were analyzed with SPSS for Windows (version 27.0). Using a 95% confidence interval, Kappa was calculated to evaluate the reproducibility of each question between the two applications (54). Additionally, the intraclass correlation coefficient (ICC) and its 95% confidence interval (CI) were calculated to verify the total reproducibility (reliability) (55). The sum of all positive questions was compared between the first and the second times the questionnaire was administered. The criteria for agreement was as follows: 0.0–0.20 (poor), 0.21–0.40 (fair), 0.41–0.6 (moderate), 0.61–0.8 (substantial), and 0.81–1.0 (almost perfect) (56). Internal consistency was calculated with Cronbach's alpha, using all initial and follow-up questions of the translated version. Significance level was set at 5% for all tests.

## Results

The Brazilian Portuguese version of the PAR-Q+ had excellent internal consistency with a Cronbach's alpha of 0.993. In terms of reproducibility, out of the 48 items of the questionnaire, 45 (93.8%) had an almost perfect agreement between the first and second applications, and three (6.2%) had a substantial agreement (follow-up questions 2a, 5e, and 8b). The translated version of the questionnaire had a good to excellent general reproducibility (ICC = 0.901, 95% CI: 0.887–0.914). Specifically, as shown in Table 1, every one of the seven general health questions of the questionnaire had an almost perfect agreement.

**Table 1:** Kappa value for each general health question between two applications of the Brazilian version of the PAR-Q+.

**General health question**	**Agreement between applications**
1	0.949
2	0.915
3	0.927
4	0.950
5	0.976
6	0.882
7	0.904

**Figure 1:**
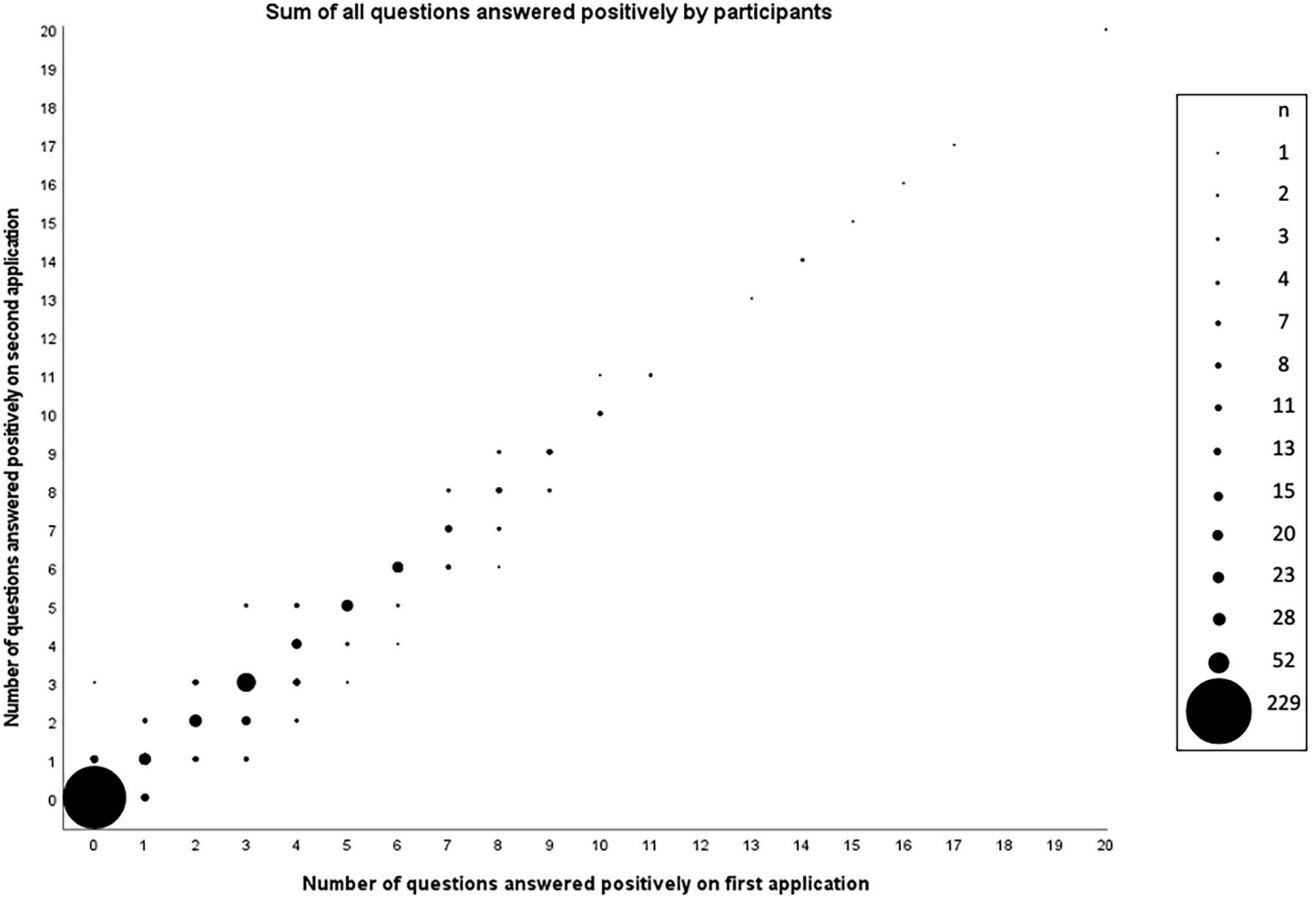
Number of questions answered positively by participants (*n* = 493) in the first and second applications of the Brazilian Portuguese version of the PAR-Q+.

The maximum of questions answered positively was 20. A total of 405 (82.2%) participants provided the same answer to every question they answered both times they completed the questionnaire. Out of those, 229 answered negatively to all questions. For those individuals who did not have the same answer for all questions in both applications, 62 had one different answer, 16 responded two questions with different answers, and 10 had three answers that did not match. Figure 1 shows the comparison of the sum of questions answered positively between the first and the second administrations of the questionnaire.

The time reported to answer the PAR-Q+ in Brazilian Portuguese was 4.4 ± 2.3 min. After answering the questionnaire, a few participants provided their opinion about their comprehension of the questionnaire. Two individuals taking medication reported uncertainty about how to answer the following question: Do you have difficulty controlling your condition with medications or other physician-prescribed therapies? (Answer **NO** if you are not currently taking medications or other treatments). Since their health conditions were under control, these individuals received clarification from the researcher, explaining they should answer negatively. No other concern was raised by any participant.

Specifically, the subsample of children and adolescents had 46 questions (95.8%) presenting an almost perfect agreement and two questions (4.2%) presenting a substantial agreement: follow-up items 1 and 5e. The group of adults also had 46 items (95.8%) with an almost perfect agreement, and two items (4.2%) with a substantial agreement, namely follow-up questions 7c and 8b. In the subsample of older adults, 45 questions (93.8%) had an almost perfect agreement and three questions (6.2%) had a substantial agreement: follow-up items 4a, 5a, and 6. All age groups had excellent internal consistency. The group of children and adolescents had a good general reproducibility, and the other two subsamples, adults and older adults, had a good to excellent total reproducibility. The time reported to answer the questionnaire, the internal consistency, as well as the general reproducibility for each one of the age groups are presented in Table 2.

**Table 2:** Time to complete the questionnaire, internal consistency, and total reproducibility according to each age group of individuals to validate the Brazilian Portuguese version of the PAR-Q+.

	**Children and adolescents (5–17 yr)**	**Adults (18–64 yr)**	**Older adults (65–93 yr)**
Time to complete (mean ± standard deviation)	4.0 ± 2.2 min	4.1 ± 2.6 min	5.0 ± 1.8 min
Internal consistency (Cronbach's alpha)	0.980	0.993	0.997
Total reproducibility (ICC; 95% CI)	0.819 (0.766–0.866)	0.905 (0.888–0.921)	0.885 (0.843–0.920)

## Discussion

The Brazilian version of the PAR-Q+ showed strong reproducibility, with all items demonstrating an agreement between substantial and almost perfect in the whole sample, as well as in each age group. This strong reproducibility is similar to the one presented by the sample of Spanish speakers, which also had all questions in these categories of agreement (57).

The PAR-Q+ was initially developed in two languages, English and French, since it was created in Canada, which is a bilingual country (58, 59). To date, the questionnaire has been officially translated into Spanish, and multiple other translation processes are in progress (57). The study about the Spanish version analyzed the participants as a single group, which had 47 items presenting an almost perfect agreement and only one presenting a substantial agreement. In the whole sample of the Brazilian study this proportion was slightly different, with 45 items presenting an almost perfect agreement and three presenting substantial agreement. There are a number of potential explanations for this variation. The Brazilian Portuguese document was validated with almost triple the sample size than the Spanish version (177 participants), and with some individuals much younger and others much older than the participants in that study (13–85 years old). Also, most individuals in the Brazilian version answered the questionnaire online. Both of these factors could be considered strengths of the current validation, given that the larger age range increases the representativeness of the population, while the online application allows for a more widespread application. It is also possible, however, that these aspects led to a couple of answers being less consistent, which could explain the slightly higher number of questions below an almost perfect agreement. However, when providing feedback about their understanding of the questionnaire, other than two individuals requiring further clarification about one question, no additional concerns were raised.

For the whole sample and for each age group, all of the seven initial questions of the Brazilian version had an almost perfect agreement. However, it is possible that some participants did not pay full attention to all follow-up questions when answering the questionnaire for the second time. This may be a factor for those who answered the PAR-Q+ online by themselves, without the presence of a health/fitness professional. According to Kung et al. (60), a low rate of inattentive answers is expected in any research based on survey responses, and this rate can be higher if there is little or no incentive for respondents to complete a survey. Although our participants voluntarily accepted to participate in the study and received a sound and thorough explanation about the research's importance and how to proceed, they were not financially compensated. Additionally, according to Schneider et al. (61), who examined self-administered and internet-based questions on quality of life, some individuals may provide careless responses when there is a lack of personal, face-to-face interaction. This is supported by Meade and Craig (62), who pointed out that the distance from the respondent to the professional in charge of the questionnaire can lead to less accountability when completing the survey. An additional possible cause of some level of inattention is the need to repeatedly answer the same questionnaire in a short period of time (63, 64). This could have been a factor in the present study, since the validation process required participants to answer the same questionnaire twice, seven to 14 days apart. However, in real-life situations, this repetitive process will not be necessary to start or increase participation in physical activity, as per the questionnaire guidelines individuals will only have to respond once within any 12-month period, unless there is a change in their health conditions. Furthermore, in their validation study of the International Physical Activity Questionnaire in 12 countries, Craig et al. (65) noted that longer questionnaires can be seen as boring and repetitive. Although the PAR-Q+ has many more questions than the previous PAR-Q, this innovative format, with the initial and the follow-up evidenced-based questions, is what makes this new screening tool unique, in providing physical activity clearance to 99% of its respondents without needing to be referred to a physician (66). Nevertheless, we showed that the Brazilian Portuguese version of the PAR-Q+, like the original document, takes approximately only 5 min to complete (19).

This questionnaire does not require that every individual who answers positively to one or more of the health general questions obtains clearance from a physician, making it a convenient screening tool in high- as well as in low- and middle-income nations. In industrialized countries, where the offer of medical services is usually sufficient for most the population, providing clearance for physical activity is often considered a time consuming and cumbersome process by physicians (67). Removing unnecessary consultations with this health professional before participating in physical activity or in a fitness appraisal is especially important in lower-income countries like Brazil, since due to social inequities in health, a large number of individuals have limited access to medical professionals (68). In fact, there is a need for low-cost and accurate self-assessment tools related to physical activity that can be utilized around the world in different cultures and ethnic groups (69). Specifically, effective screening provides a significant contribution to maximize physical activity engagement at the population level (16). Accordingly, having the PAR-Q+ properly translated and culturally validated to the Brazilian Portuguese language can contribute to greater numbers of individuals to safely start or increase physical activity participation.

## Limitations

While the present study has a considerable sample size, with individuals living in different locations, this cohort is not necessarily representative of the entire Brazilian population. To address this issue, participants were recruited in the most populous city in the country (São Paulo), which contains individuals from all Brazilian states. Participants were also recruited in two other major cities: Campinas and Vancouver (Canada). Another limitation was the fact that the cognitive debrief happened during the data collection instead of at a specific pre-test moment. The intention was to allow every participant to provide feedback about their understanding of the questionnaire.

## Conclusion

Based on the findings of this study it can be concluded that, overall, Brazilians of different ages, male and female, healthy or living with chronic medical conditions, had no difficulty in understanding the translated and adapted version of the questionnaire. The results also indicate that participants were able to similarly complete the Brazilian Portuguese version of the PAR-Q+ on two independent occasions, showing the strong reproducibility of the questionnaire. Altogether, these outcomes demonstrate that the PAR-Q+ in Brazilian Portuguese is a valid and reliable screening tool. It is expected that nationwide implementation of the questionnaire could allow a substantial number of Brazilians to safely engage in more physical activity participation, as well as in fitness assessments, providing ways to enhance wellness and to contribute toward the prevention and management of chronic diseases in this population.

## Data Availability Statement

The raw data supporting the conclusions of this article will be made available by the authors, without undue reservation.

## Ethics Statement

The studies involving human participants were reviewed and approved by the Research and Ethics Board of the University of British Columbia. Written informed consent to participate in this study was provided by the participants' legal guardian/next of kin.

## Author Contributions

JS and PO designed the study. JS, MT, BS, ED, JC, and MM were responsible for data collection. JS and MT were responsible for statistical analyses. JS drafted the manuscript. PO, MT, BS, ED, EF, RR, SB, JC, PdS, MM, and DW critically revised the manuscript. All authors contributed to the article and approved the submitted version.

## Conflict of Interest

The authors declare that the research was conducted in the absence of any commercial or financial relationships that could be construed as a potential conflict of interest.

## Publisher's Note

All claims expressed in this article are solely those of the authors and do not necessarily represent those of their affiliated organizations, or those of the publisher, the editors and the reviewers. Any product that may be evaluated in this article, or claim that may be made by its manufacturer, is not guaranteed or endorsed by the publisher.
